# Phenotypic and Genotypic Characterization of a Highly Virulent *Erysipelothrix rhusiopathiae* Strain

**DOI:** 10.1155/2024/5401707

**Published:** 2024-07-26

**Authors:** Dun Zhao, Yuli Hu, Haichao Wu, Zhao Feng, Chengcai Hu, Huican Hu, Yang Liu, Wen Sun, Xinglong Yu

**Affiliations:** ^1^ College of Veterinary Medicine Hunan Agricultural University, Changsha, China; ^2^ R&D Center Sinopharm Animal Health Co., Ltd., Wuhan, China

## Abstract

*Erysipelothrix rhusiopathiae* is responsible for erysipelas infection in pigs. Outbreaks of *E. rhusiopathiae* have increased in several countries, including China, over the past two decades. An *E. rhusiopathiae* strain (ML101) was isolated and characterized from dead pig tissue sample collected from a farm experiencing an outbreak of *E. rhusiopathiae*, which was responsible for the deaths of 146 sows and 308 fattening pigs within a week. Spleen swelling, gastric and bladder mucosa bleeding, and submandibular lymph node swelling and bleeding were observed through necropsy. ML101 was identified as serotype 1a via molecular analysis and immunological assays. Studies in mice demonstrated that the minimal lethal dose per animal was less than 10 colony-forming units (CFU). Notably, the minimal lethal dose in piglets was also less than 10 CFU, which is lower than that of any *E. rhusiopathiae* strain reported to date. The challenged piglets showed typical acute erysipelas symptoms, such as pyrexia, hemorrhage, depression, complete inappetence, reddening, and purpling skin on the buttock. Evidence of efficient horizontal transmission was observed, as healthy pigs were infected and died when cohoused with challenged piglets. Whole-genome sequencing revealed that ML101 contained a 77 kb genomic island (GI), carrying a Tn*916* transposon and a multidrug resistance gene cluster (*aadE-apt-spw-lsa*(*E*)-*lnu*(*B*)-*aadE-sat4–aphA3*). A retrospective analysis of *E. rhusiopathiae* isolates via PCR indicated that the GI has been widely distributed since 2010, when outbreaks were more frequently reported in China. This study demonstrated that the highly virulent *E. rhusiopathiae* is responsible for the erysipelas outbreak and indicates that relevant genes located within the transmissible genetic elements may play roles in virulence. Therefore, epidemiological monitoring needs to be emphasized to better prevent and control erysipelas in the swine industry, and live attenuated vaccines should be used with caution.

## 1. Introduction

*Erysipelothrix* (*E*.), belonging to the family Erysipelotrichaceae, order Erysipelotrichales, class Erysipelotrichia, and phylum *Firmicutes* [1], are facultative anaerobic, Gram-positive, rod-shaped bacteria that do not form spores. Traditionally, *Erysipelothrix* spp. are thought to have more than 20 serotypes in two species: *E. rhusiopathiae* (serotypes 1a, 1b, 2, 4–6, 8, 9, 11, 12, 15–17, 19, 21, and N) and *E. tonsillarum* (serotypes 3, 7, 10, 14, 20, 22, 23, and 24–26). Two other serotypes, 13 and 18, have been suggested as other *Erysipelothrix* species strains 1 and 2, respectively [2]. The genome size of *E. rhusiopathiae* ranges from 1,770,411 to 1,945,689 bp, with a G + C content of approximately 36.5% [3]. *E. rhusiopathiae* is a zoonotic pathogen that causes erysipelas in a wide variety of animal species and erysipeloids in humans, particularly after occupational exposure, such as contact with contaminated pork or pork-derived products [4]. Among the affected animal species, swine is the most economically important. There are three clinical erysipelas forms: the acute form (with sudden death or pink, red, or purple urticarial skin lesions), the subacute form with clinically less severe signs, or the chronic form with arthritis or vegetative valvular endocarditis. Despite more than 10 serotypes of *E. rhusiopathiae*, only serotypes 1a, 1b, and 2 have been prevalent in pigs globally, according to epidemiological studies conducted in the last two decades [4]. *E. rhusiopathiae* is an old swine disease-causing pathogen that can be traced to 1882 [4]. Owing to vaccines, antibiotics, and housing management in the pig industry, *E. rhusiopathiae* was well-controlled for several decades until around 2010, after which increasing outbreaks were reported in many countries, including China [5], Japan [6], and the USA [7].

Vaccines that prevent erysipelas include live attenuated, inactivated, autogenous, and subunit vaccines [4]. Traditionally, these vaccines have been effective in controlling *E. rhusiopathiae* infections. However, increasing outbreaks indicates that mutations in the immunogens of wild strains are, to some extent, responsible for vaccination failure [7, 8]. Among them, cell wall-anchored Spa is the most important antigen. Three types of Spa, namely, SpaA, SpaB, and SpaC, were identified in various serotypes. Amino acid sequence similarities between different Spas range at 61%–67%. Cross-protection can be induced by each other, but SpaC elicits the most robust cross-immunity, such as conferring protection to 90%–100% mice after challenge with SpaB- or SpaC-carrying strains in various serotypes and to 100% pigs after challenge with serotype 1a, SpaA-carrying virulent strain Fujisawa [9]. Although SpaA induces less cross-protection to heterogenous Spa-carrying strains, complete protection can be elicited to homologous SpaA-bearing strains belonging to serotypes 1a and 2, all of which contain SpaA only [7, 10]. This might be why currently licensed subunit vaccines are based on SpaA. SpaA plays a pivotal role in pathogenesis, such as adhesin to attach host cells [11]. On the other hand, SpaA-specific antibodies facilitate phagocytosis and clearance of the bacteria in macrophages by opsonization [12]. For the roles of SpaA in pathogenesis and protective immunity, complete or partial SpaA-encoding sequences were determined to type isolates and ascertain whether the vaccine was a suitable match for prevalent strains. Diverse SpaA variants have been identified in clinical isolates worldwide [7, 8, 13].

Other surface-exposed proteins, such as RspA, GAPDH (glyceraldehyde-3-phosphate dehydrogenase), and CbpB, participate in adhesion and inducing opsonic antibodies for phagocytic killing by macrophages [4]. Capsule is a key virulence factor by providing resistance to phagocytosis and facilitating cell survival. Enzymes such as neuraminidase cleaving host sialic acid from sialo-glycoconjugates, leading to widespread vascular damage, and hyaluronidase facilitating the dissemination of the bacteria into tissues play roles in pathogenesis [12].

The use of attenuated vaccines in several countries has raised concerns regarding the recurrence of virulence. Serotyping and molecular analysis of isolates confirmed vaccine strain-related diseases in pigs [14]. Further studies revealed recombination among different field strains [15].

Antibiotic-resistant strains have been increasingly reported over the years. For example, after treatment of bacterial respiratory diseases and diarrhea in pigs with dihydrostreptomycin and oxytetracycline in Japan, there was a tendency of increasing resistance to these two antibiotics from 1988 to 1998 [16]. Furthermore, the percentage of oxytetracycline-resistant strains increased from 53.3% (1988–1998) to 72.1% (1994–2001) [17]. Abuse of antibiotics is not the only reason for widespread resistance [18]; the horizontal transfer of drug-resistance genes might play an important role [19]. Ozawa et al. [17] reported that a Tn*916*-like transposon containing *tetM* could be detected via PCR in 77.6% (38/49) of tetracycline-resistant *E. rhusiopathiae* strains. Recently, a Tn*916* transposon with multidrug resistance genes was identified in the strains ZJ, GYBY-1, B18, B52, B2, and SE25 isolated in China [5, 20, 21]. In addition, a plasmid-borne macrolide and lincosamide resistance-conferring gene was identified [22]. Thus, issues regarding the vaccine and antibiotic resistance of *E. rhusiopathiae* strains make erysipelas difficult to control.

An animal challenge is one of the most commonly used methods to directly determine the virulence of strains. It is well-known that mice are extremely susceptible to the *E. rhusiopathiae* challenge, and the 50% lethal dose in mice can be as low as 10 colony-forming units (CFU) [2]. However, approximately 10^6^ CFU of *E. rhusiopathiae* is needed to infect pigs to induce typical diseases for most of the reported virulent strains [10, 13, 23, 24]. Thus, mouse models do not always accurately reflect the virulence of the *E. rhusiopathiae* strain.

Despite extensive analysis of *spaA* sequences, antibiotic resistance phenotypes, antibiotic resistance genes, the recovery of attenuated vaccine-related strains from diseased pigs, and the determination of virulence in mice, the reasons for the increasing outbreaks remain unclear.

To investigate the reasons for the recent outbreaks of *E. rhusiopathiae*, this study aimed to test the virulence of the here-defined strain, ML101, in mice and piglets, in addition to the resistance to commonly used antibiotics. Furthermore, we investigated the mechanisms underlying antibiotic resistance and increasing virulence at the genomic level.

## 2. Materials and Methods

### 2.1. Bacterial Strains and Culture Conditions

An intensive breeding pig farm in Miluo City, Hunan province, had approximately 650 sows, and their 2,000 offspring at varying ages were housed in the same site. The sows were vaccinated against viral diseases, including swine classic fever, foot-and-mouth disease, porcine pseudorabies, and Japanese encephalitis. The fattening pigs were immunized with the vaccines against swine classic fever, foot-and-mouth disease, and porcine pseudorabies. None of the pigs on this farm was immunized with bacterial vaccines. In 2010, an outbreak leads to 146 sows (146/650, 22.46%) and 308 fattening pigs (308/2000, 15.4%) death within 4 days. The farm owner recorded that some pigs were consistently lying down and reluctant to move and presented with fever, loss of appetite, and typical red or purple-raised firm rhomboids on the skin before death. Necropsy performed after death showed numerous bloody bubbles in the tracheas, enlarged spleens, and hemorrhages in the gastrointestinal tracts, kidneys, and bladders of affected pigs. Two dead sows and three dead fattening pigs were necropsied, and their heart, liver, kidney, lung, submandibular and mesenteric lymph nodes, and spleen were subject to bacterial isolation in our laboratory.

The 35 tissue samples were streaked onto sheep blood agar plates (Bio-caring, Jiangmen, China), followed by aerobic culture at 37°C for 48 hr. Single colonies were streaked onto agar for three cycles to obtain pure cultures. A colony was randomly selected, designated ML101, and further purified by colony picking for three cycles. This strain was cultured in tryptic soy broth (TSB) medium (Becton, Dickinson and Company, Franklin Lakes, NJ, USA) supplemented with 10% fetal bovine serum (Tianhang, Hangzhou, China) at 37°C and shaken at 180 rpm for 36 hr. A growth curve was constructed as follows: 1% of overnight culture was inoculated into fresh TSB medium supplemented with 10% fetal bovine serum, and bacterial counting was performed on tryptic soy agar (TSA) agar (Becton, Dickinson and Company) supplemented with 10% fetal bovine serum after 10-fold serial dilutions at 2-hr intervals. Fresh cultures were stained with a Gram staining kit (Hangzhou Microbial Reagent, Hangzhou, China) according to the manufacturer's instructions. The previously preserved 40 *E. rhusiopathiae* strains (*Supplementary table 1*) were isolated from clinical samples sent to our laboratory with sheep blood agar similarly and cultured in TSB supplemented with bovine serum.

### 2.2. PCR

To identify ML101, 16S rDNA was amplified as previously described [25] followed by sequencing and Basic Local Alignment Search Tool (BLAST) analysis. Based on these results, a set of serospecific primers [26] was used to determine the serotype of *E. rhusiopathiae* ML101. To screen piglets for the bacterial challenge, DNA in the blood samples and anal and tonsil swabs were extracted with the DNeasy Blood and Tissue Kit (Qiagen, Dusseldorf, Germany) to detect *E. rhusiopathiae* [27], while viral DNA and RNA were extracted with MiniBEST Viral RNA/DNA Extraction Kit (Takara, Dalian, China) from the samples to detect swine classic fever virus [28, 29], porcine reproductive and respiratory syndrome virus [30], foot-and-mouth disease virus [31], porcine pseudorabies virus [32], and porcine circovirus 2 [33]. cDNA was synthesized by PrimeScript™ IV 1^st^ strand cDNA Synthesis Mix (Takara, Dalian, China) according to the manufacturer's instructions. To detect genomic island (GI) in *E. rhusiopathiae* strains, sets of primers targeting endonuclease, recombinase, and other specific regions were designed with the Primer Premier version 5.0 [34]. All primer sets are listed in *Supplementary table 2*, and PCR reactions performed in this study were single reactions.

### 2.3. Enzyme-Linked Immunosorbent Assay

To confirm the piglets used for challenge were sero-negative, *E. rhusiopathiae*-specific antibodies were detected with the ELISA kit, CIVTEST SUIS SE/MR (HIPRA, Girona, Spain), according to the manufacturer's instructions.

### 2.4. Serotyping Assay

The ML101 serotype was determined as described previously with minor modification [35]. Briefly, ML101 was inoculated to fresh TSB medium supplemented with 10% bovine serum and cultured at 37°C and 180 rpm for 36 hr before adding 1% formaldehyde to inactivate the bacteria for 24 hr. Subsequently, the bacteria were recovered via centrifugation at 1,500x*g* at 25°C for 30 min. After discarding the supernatant, the bacteria were washed thrice with 0.5% formaldehyde in 0.01 M phosphate-buffered saline (PBS, pH 7.2). The resultant bacteria were suspended in 2 mL ddH_2_O and autoclaved at 121°C for 1 hr. After cooling to 25°C, the supernatant was collected via centrifugation (1,500x*g*) at 25°C for 15 min. The resulting supernatant was used for serotype determination using standard agar diffusion against serospecific sera provided by the China Institute of Veterinary Drug Control. Agar was prepared by dissolving agar powder in 0.01M PBS (pH 7.2) at a final concentration of 1.2% (w/v). The dissolved agar was poured into Petri dishes at a depth of 3 mm and allowed to solidify at 25°C. Wells (3 mm in diameter) were made with a hole punch, and the interwell space was 4 mm. Serospecific sera were added to the central wells, while the test antigen, PBS, and the standard antigens were added to the surrounded wells. After incubation at 37°C for 24 hr, the results were recorded.

### 2.5. Biochemical Testing

After the molecular identification of strain ML101, biochemical tests were performed using Biolog® GP microplate panels (Biolog, Hayward, CA, USA). The bacterial suspension of the ML101 used for these assays was cultured until it reached the late log phase.

### 2.6. Antimicrobial Susceptibility Testing

Minimal inhibitory concentrations (MICs) of penicillin, streptomycin, ampicillin, amoxicillin, florfenicol, ceftriaxone, levofloxacin, tetracycline, enrofloxacin, and vancomycin (Sinopharm, Beijing, China) were determined by broth microdilution method recommended by the Clinical and Laboratory Standards Institute [36]. The ML101 colonies were suspended with fresh cation-adjusted Mueller–Hinton broth supplemented with lysed horse blood medium (Shifengbiol, Shanghai, China) to a density of 0.5 McFarland standard followed by 20-fold dilution. A 10 *μ*L of the dilution was added to 100 *μ*L of antibiotics agent solution before incubation at 35°C for 24 hr. The lowest concentration of an antimicrobial agent that visibly inhibited bacterial growth was determined as the MIC value. *S. pneumoniae* ATCC® 49619 was used as the quality control strain.

### 2.7. Animal Experiments

#### 2.7.1. Determination of the Minimal Lethal Dose (MLD) in Mice

Fifty 3–4-week-old Institute of Cancer Research (ICR) female mice were randomly divided into five groups, each containing 10 mice. Four of these groups were intraperitoneally injected with 100 *μ*L of 0.9% isotonic sodium chloride solution-diluted bacteria containing 5, 10, 100, and 1,000 CFU. Another group of mice was injected with 0.9% isotonic sodium chloride solution and used as a blank control. Each group of mice was housed independently. The mice were observed daily for 14 days, and deaths were recorded daily.

#### 2.7.2. Determination of the MLD in Pigs

To determine the MLD of ML101 in pigs, 7–9-week-old Duroc × Landrace × Yorkshire pigs free of *E. rhusiopathiae*, as detected via PCR, as well as the absence of specific antibodies upon ELISA, were used. A total of 30 pigs were divided into six groups, each containing five pigs. Five of these groups were intramuscularly injected with approximately 10, 1 × 10^3^, 1 × 10^5^, 1 × 10^7^, or 1 × 10^9^ CFU/pig, and the final group injected with 0.9% isotonic sodium chloride solution was used as a healthy control. All pigs were observed twice daily, and clinical manifestations and deaths were recorded. The dead pigs were necropsied, and the lymph nodes, spleens, livers, kidneys, hearts, and blood samples were used for bacterial isolation with sheep blood agar, while lymph nodes, spleen, liver, lung, and kidneys were used for pathological analyses.

#### 2.7.3. Comingling Infection

Five healthy pigs lacking *E. rhusiopathiae*- and *E. rhusiopathiae*-specific antibodies were housed together. Another five healthy pigs were intramuscularly injected with 10^5^ CFU of the ML101 strain and housed with the five healthy pigs. These five pigs were observed daily, and deaths were recorded.

### 2.8. Histopathological Examination

The tissue samples were fixed in 4% paraformaldehyde solution at 25°C for 48 hr, and histological sections were prepared for subsequent hematoxylin and eosin staining at Wuhan Servicebio Technology Co., Ltd. as described elsewhere [37].

### 2.9. Whole-Genome Sequencing

Genomic DNA for whole-genome sequencing was extracted using the DNeasy Blood and Tissue Kit (Qiagen, Dusseldorf, Germany) according to the manufacturer's instructions. The whole-genome sequence of the ML101 strain was determined at Shanghai Majorbio Bio-Pharm Technology Co., Ltd., using single-molecule sequencing technology after the construction of the 8–10 k insertion library, as described previously [38].

### 2.10. Biological Information Analysis

#### 2.10.1. *spaA* Genotyping

SpaA-encoding gene in each *E. rhusiopathiae* genome sequence was extracted. Nucleotide and amino acid sequence alignments were performed with DNAStar software package (DNASTAR Inc. Madison, Wisconsin, USA).

#### 2.10.2. GI Analysis

Mauve [39] was used to conduct a colinearity analysis to compare the ML101, the live vaccine strain used in China, G_4_T_10_, and the extensively researched virulent strain SE38, which can cause the death of a group of piglets 6 days postchallenge with 5 × 10^8^ CFU/pig [24]. The identified large insertion sequence was input into OriTfind [40] to search for transposons. The virulence genes were predicted by searching a virulence factor database [41]. Easyfig version 2.2.5 [42] was used to compare the GI with those in other bacterial strains.

#### 2.10.3. Genome-Wide Single-Nucleotide Polymorphisms (SNPs) Analysis

Whole-genome sequences of *E. rhusiopathiae* strains isolated from pigs (*Supplementary table 3*) were downloaded from GenBank (https://www.ncbi.nlm.nih.gov/nuccore/). These genomes were compared with the ML101 using Mauve [39] with the default parameters. SNPs were exported, and all SNPs in the same strain were concatenated into a single sequence to be used for the construction of a phylogenetic tree using the maximum likelihood method based on the Tamura-Nei model with MEGA6 [43, 44].

### 2.11. Statistical Analysis

GraphPad Prism version 6.0 (GraphPad Software, USA) was used for statistical analysis. Two-way analysis of variance was used to analyze the average survival time postchallenge. Errors were expressed as mean ± standard deviation unless otherwise mentioned. *P* values less than 0.05 and 0.01 were considered significant and highly significant, respectively.

### 2.12. Accession Numbers

The whole-genome sequence of the ML101 strain was deposited in GenBank (https://www.ncbi.nlm.nih.gov/nuccore/) under accession number NZ_CP029804.

## 3. Results

### 3.1. Isolation and Identification of *E. rhusiopathiae* ML101

After incubation at 37°C for 48 hr, small light-white circular colonies with smooth edges were observed on the agars streaked with the organs from the necropsied pigs (*Supplementary figure 1*). Microscopic observation of ML101 in the mid-log phase (*Supplementary figure 2*) revealed Gram-positive bacteria (*Supplementary figure 3*). The 16S rDNA sequence of the strain was amplified via PCR (*Supplementary figure 4*) and determined via Sanger sequencing, suggesting that the strain was *E. rhusiopathiae*, as shown via BLAST analysis (*Supplementary sheet 1*). Furthermore, biochemical tests showed positive results for glucose, lactose, galactose, mannitol, and maltose fermentation and negative results for fructose, sucrose, arabinose, salicin, and xylose fermentation. Additionally, nitrate and H_2_S tests yielded positive results while Voges–Proskauer, uricase, indole, citrate, oxidase, and methyl red tests (*Supplementary table 4*). The ML101 serotype was further determined using serospecific PCR (*Supplementary figure 4*) and agar diffusion assays (*Supplementary figure 5*). Both assay results showed that ML101 was a 1a serotype. The results of MICs of antibiotics against the ML101 showed as follows: penicillin (<0.015 *μ*g/mL, susceptible), amoxicillin (<0.015 *μ*g/mL), ampicillin (0.06 *μ*g/mL, susceptible), enrofloxacin (1 *μ*g/mL, intermediate), levofloxacin (0.5 *μ*g/mL), florfenicol (1 *μ*g/mL), streptomycin (>512 *μ*g/mL), tetracycline (4 *μ*g/mL), and vancomycin (8 *μ*g/mL) and ceftriaxone (<0.015 *μ*g/mL) (*Supplementary table 5*).

### 3.2. ML101 Is a Highly Virulent *E. rhusiopathiae* Strain

Considering that mice are sensitive to *E. rhusiopathiae*, we first tested the virulence of ML101 in ICR mice. All mice challenged with 1,000, 100, or 10 CFU died, whereas 80% of the mice challenged with 5 CFU died postchallenge, and ML101 was recovered from their organs (data not shown), showing that the MLD in ICR mice was as low as 10 CFU (*Supplementary table 6*).

**Figure 1:**
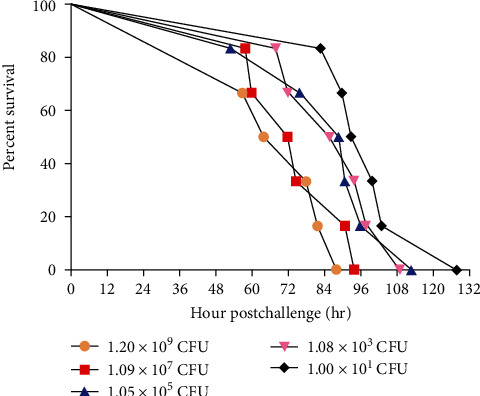
Survival curve of piglets postchallenge with the ML101 strain at different doses. The average survival time of piglets challenged with 10 CFU was significantly longer than that of those challenged with 10^9^ CFU (*P* < 0.05).

**Figure 2:**
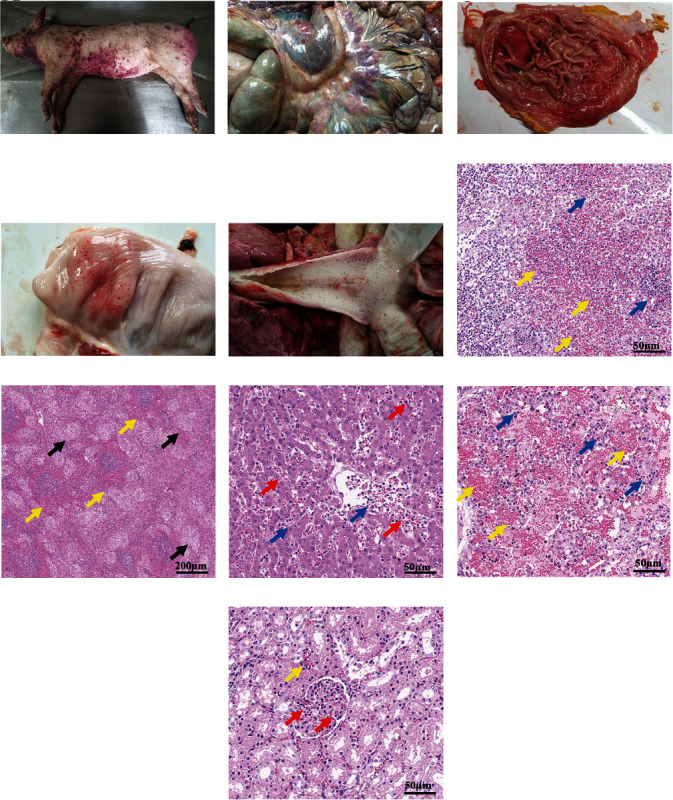
Macroscopic and microscopic examination of ML101-infected piglets. Large papules and plaques were observed on the body surface of the infected pigs (a), with varying degrees of hemorrhage in the mesenteric lymph nodes (b), gastric mucosa (c), bladder mucosa (d), and bubbles in the trachea (e). Hematoxylin and eosin (H&E) staining of the lymph node (f), spleen (g), liver (h), lung (i), and kidney (j) sections of infected piglets: Hemorrhage is indicated by yellow arrows; hyperemia in the liver and kidney is indicated by red arrows; necrosis in the spleen is indicated by black arrows; and infiltration of inflammatory cells in the lymph, liver, and lung is indicated by blue arrows.

We similarly determined MLD in piglets with dosage ranges from approximately 10^1^ to 10^9^ CFU. Anorexia, weakness, and an increase in body temperature were observed within 24 hr postchallenge. Notably, all these challenged piglets died 48–132 hr postinfection, showing that the MLD of ML101 in piglets was less than 10 CFU (Figure 1). The average survival time after the challenge was dose-dependent, showing that the survival time increased as the challenge dose decreased (Figure 1). Among them, the average survival time of piglets challenged with 10 CFU was significantly longer than that of those challenged with 10^9^ CFU (*P* < 0.05). ML101 infection was confirmed by recovering the bacteria from the blood of dying or dead piglets rather than that of live piglets 12, 24, 36, and 48 hr postchallenge, regardless of the infection dose (data not shown). Erythema was commonly observed at the injection site at 12 hr postinfection, followed by reddening, purpling, and expansion to form a large lump (19/25) during macroscopic examinations (Figure 2(a), Table 1). To characterize the clinical lesions caused by the ML101 strain, all 25 dead pigs from the five groups were necropsied. As summarized in Table 1, swelling and bleeding were the two most common features seen in the organs. For example, swelling of the mesenteric lymph nodes, bleeding of the gastric and bladder mucosa, and bubbles in the trachea and/or bronchus were observed in dead piglets (Figures 2(b), 2(c), 2(d), and 2(e)). The organs were then subjected to pathological section analysis to further investigate the pathogenic changes. The results showed that hemorrhage and inflammatory infiltration were commonly seen in the lymph nodes, spleen, liver, lungs, and kidney compared with those in the controls (Figures 2(f), 2(g), 2(h), 2(i), and 2(j)). Additionally, fluid from the lungs, kidney tubule necrosis, and white pulp atrophy were observed (Figures 2(i) and 2(j)).

To determine whether ML101 is contagious, as observed on the farm, five piglets were artificially infected with 10^5^ CFU before comingling with five healthy piglets. As expected, artificially infected piglets died at 75 hr postinfection, similar to the results of the MLD determination assay. On the other hand, the five sentinel piglets were showed similar clinical signs and lesions to those artificially infected piglets and died 140 hr after comingling, suggesting the ML101 is highly contagious.

### 3.3. Whole-Genome Sequence Analysis of ML101

To investigate the molecular mechanisms underlying its high virulence, the whole-genome sequence of ML101 was determined. The whole genome, with a GC content of 36.4%, was 1,854,248 bp in length, encoding 1,699 proteins, as predicted via genome annotation; these findings are similar to those of other *E. rhusiopathiae* strains whose whole genomes have been published [3]. Genotyping the *spaA* revealed that methionine and isoleucine at amino acid positions 203 and 257 of the SpaA, belonging to the Met203/Ile257 variant type (Table 2).

**Figure 3:**
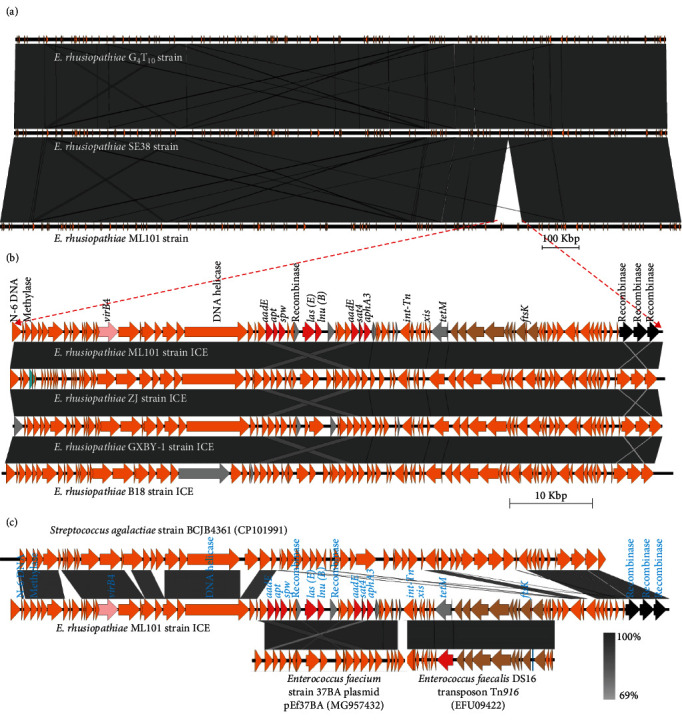
Colinearity analysis of the complete or partial ML101 genome with reference sequences. (a) Comparison of the complete ML101 G_4_T_10_ and SE38 genome. (b) Comparison of the identified GI in the ML101 genome within the ZJ, GXBY-1, and B18 genomes. (c) Comparison of GI in the ML101 genome with non-*E. rhusiopathiae* sequences. Tn*916* transposon prototype from *E. faecalis*, plasmid pEf37BA from *E. faecium*, and partial genome of *Streptococcus agalactiae*.

To understand the pathogenic mechanisms of ML101, we first searched for virulence genes in the ML101 genome. We found that the genome contained well-known virulence-related genes, including genes encoding capsule synthesis enzymes, SpaA, and RspA as summarized in *Supplementary table 7* [12, 45, 46, 47, 48, 49]. A comparative genome analysis was performed between ML101, G_4_T_10_, which is an attenuated vaccine strain that has been widely used in the pig industry in China for several decades, and SE38, a virulent strain in pigs. A total of 232 SNPs were identified, while only 12 sites were ML101-specific (*Supplementary sheet 2*). These SNPs were in coding regions of G5 domain-containing protein (DM789_RS09250, seven SNPs, leading to premature at aa 117), 16S rRNA (DM789_RS00380, 1 SNP), adenine daminase C-terminal domain-containing protein (DM789_RS04440, one SNP), 23S rRNA, (DM789_RS05365, two SNPs), and site-specific integrase (DM789_RS07070, one SNP). Insertion and deletion analysis showed that three and one deletion(s) compared with G_4_T_10_ and SE38, respectively, but all these four sequences are encoding transposases in different families. ML101-specific insertions include two transposase-encoding genes (DM789_RS01195 and DM789_RS04875), sequence encoding “PEVKPEEKPEVKPEEK” in C-terminus of a cell wall-anchored (containing an LPKTG motif in C-terminus) discoidin domain-containing protein (M789_RS02905), and a 77,230 bp insertion that was not present in G_4_T_10_ and SE38 (Table 3, Figure 3(a)). However, this insertion was found in B18, GXBY-1, and ZJ strains, showing sequence identity higher than 99%. Mutation, insertion, and deletion lead to premature or frameshift-making pseudo genes in each strain, such as *tetM* in the ML101, which results in a frameshift at aa 261 and a premature frameshift-making at aa 289 rather than a functional full length of 640 aa. This 77-kb insertion contained 84 predicted reading frames, including a Tn*916* transposon similar to that found in *Enterococcus faecalis*, as analyzed via OriTfinder (Figure 3(b)), a multidrug resistance gene cluster (*aadE-apt-spw-lsa* (*E*)-*lnu* (*B*)-*aadE-sat4–aphA3*) similar to that identified in *Enterococcus faecium*-origin plasmid, and the remaining sequence was found in phylum Firmicutes, including *Streptococcus* (e.g., *S. agalactiae*, *S. pneumoniae*, *S. pseudoporcinus*, *S. pyogenes*, and *S. dysgalactiae*), *Clostridioides difficile* (>95% identity and >70% coverage), and *E. faecalis* (>94% identity and >60% coverage), as well as *Anaerococcus*, *Ezakiella*, *Fastidiosipila*, *Finegoldia*, *Granulicatella*, *Helcococcus*, and *Peptoniphilus* (>95% identity and coverage ranging 60%–80% for these seven genera) as showed by BLAST analysis excluding *E. rhusiopathiae* (*Supplementary sheet 3*).

### 3.4. GI Is Widely Distributed among *E. rhusiopathiae* Strains

We conducted a molecular epidemiological analysis of the GI. Results showed that 92.5% (37/40) of the strains isolated since 2010 contained GI, except for one serotype 1a strain isolated from Hengyang city in Hunan province and two serotype 2 strains from Guangdong province in 2014 (*Supplementary table 1*).

### 3.5. Phylogenetic Analysis Based on Genome-Wide SNPs

**Figure 4:**
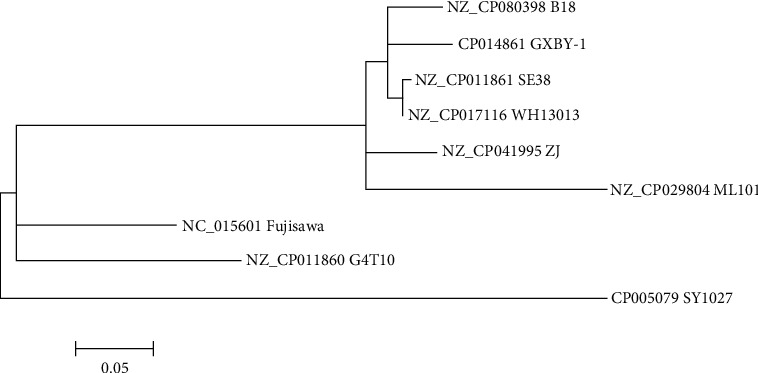
Phylogenetic tree based on genome-wide SNPs. The tree with the highest log likelihood (−3,171.3855) is shown.

A total of 899 SNPs were found after comparison among serotype 1a strains isolated in pigs, including the ML101, G_4_T_10_, SE38, B18, ZJ, GXBY-1, WH13013, SY1027, and Fujisawa (*Supplementary sheet 4*). The SNP-based phylogenetic tree shows that the ML101 is phylogenetically close to five isolates, namely, SE38, B18, ZJ, GXBY-1, and WH13013, but phylogenetically separate from SY1027 (Figure 4).

## 4. Discussion

*E. rhusiopathiae* has more than 10 serotypes, of which serotypes 1a, 1b, and 2 are globally prevalent in the pig industry [4]. Erysipelas re-emerged in China in 2010 [50, 51], and more outbreaks have been reported since then. Similarly, increasing cases and outbreaks were recorded in other pig-raising countries, such as Japan and the USA [7, 52, 53]. Health and immunological status of the animals, vaccination breakdown, changes in antimicrobial usage, and changes in environmental conditions that favored the growth of *E. rhusiopathiae*, such as diet or stress, were proposed to explain the re-emergence of erysipelas [4]. In the present study, bacterial isolation of the organs from dead pigs on the farm with an outbreak on blood agar plates revealed a serotype 1a *E. rhusiopathiae*. The MLD of the isolate was less than 10 CFU, consistent with previous studies that showed that mice are extremely sensitive to *E. rhusiopathiae* [2]. Even in the *spaA*-deletion mutant with a 76-fold reduction in virulence, the LD_50_ in mice was less than 700 CFU [54]. However, the concentrations of *E. rhusiopathiae* used to challenge pigs are typically higher than 10^6^ CFU, including strain Fujisawa (serotype 1a), 82–875 (serotype 2), SE38 (serotype 1a), and M203/I257 SpaA variant wild strain (serotype 1a) [10, 13, 23, 24]. Notably, the MLD of the ML101 strain in pigs was <10 CFU. The necropsy results of the dead pigs were also consistent with those seen in typical acute erysipelas described elsewhere [55]. Microscopic examination of the pig tissues showed hemorrhage and inflammatory infiltration, consistent with the acute form of erysipelas. These results were concordant with the observations made on the pig farm and demonstrated that the highly virulent strain was contagious.

To investigate why the ML101 strain is highly virulent in pigs, the whole genome was sequenced using next-generation sequencing technology and compared with the vaccine strain G_4_T_10_ and the virulent strain SE38 isolated in China in 2014, which caused the death of a group of pigs within 3–6 days postchallenge with 5 × 10^8^ CFU/pig [24]. The results showed that ML101-specific SNPs, insertions, and deletions seem not to relate to known virulence-associated genes (*Supplementary sheet 2*, Table 3), except for an LPXTG motif-containing discoidin domain-containing protein (DM789_RS02905) and an approximately 77-kb insertion (Table 3, Figure 4(a)).

This 77-kb insertion was also found in ZJ, GXBY-1, and B18 strains isolated in different provinces in China from 2012 to 2018 [5, 20, 21]. Although these three strains were isolated from pigs recorded as diseased or not, virulence in pigs was not identified. Sequence analysis of the 77-kb insertion revealed that it consist of a Tn*916* transposon, a multidrug resistance gene cluster (*aadE-apt-spw-lsa* (*E*)-*lnu* (*B*)-*aadE-sat4–aphA3*), and sequences similar to those found in members in phylum *Firmicutes*, most possibly *Streptococcus*, as showed by BLAST search (*Supplementary sheet 3*), suggesting the 77-kb insertion originated from interspecies recombination followed by horizontal gene transfer. *E. rhusiopathiae* is an intracellular pathogen, and resistance to intracellular oxidative burst is pivotal for survival and further replication with the cells. Capsule confers resistance of the bacteria to phagocytosis by macrophages and protects from oxidative burst damages produced by the cells [12]. These sequences encode several genes potentially related to oxidative stress responses, such as copper amine oxidase (DM789_06950) for alleviating toxicity of copper [56], RibD (DM789_06815) for riboflavin (the precursor flavin coenzymes FMN and FAD RedOx reactions) biosynthesis, which is lacking in Fujisawa strain [1], sigma-70 family RNA polymerase (DM789_06625) playing roles in virulence by regulating transcription of stress response and virulence-associated genes [57], and XRE family transcriptional regulators (DM789_06635 and DM789_06920), known to regulate virulence and oxidative stress responses of the family [38, 48]. However, whether these annotated oxidative stress response genes help the ML101 to survive and overwhelm the host remains to be investigated.

Surface proteins on the *E. rhusiopathiae*, such as SpaA, RspA, RspB, GAPDH, HP0728, and HP1472, are crucial in pathogenesis by adherence of the host cells [11, 46, 47, 54, 58). Specifically, deletion of two copies of C-terminal repeats of the SpaA affects the virulence of the SE38 strain by affecting adherence to host cells [11]. In the ML101 strain, insertion of four “PEE/VK” repeats in the C-terminal of discoidin domain-containing protein (DM789_RS02905), an LPXTG motif-containing cell wall anchored protein, might affect the virulence as that of SpaA.

Other virulence-associated genes, such as capsule synthesis enzymes [59] and *spaA* [54], were also found in ML101 (*Supplementary table 7*), similar to those reported by Yang et al. [3] who performed a comparative genome analysis, including ML101. Thus, the profile of these virulence genes and the M203/I257 SpaA variant type in strain ML101 cannot explain its higher virulence.

Antimicrobial drugs are widely applied to control bacterial diseases in the pig industry. For *E. rhusiopathiae* from pigs in China, resistance to penicillin, cefotaxime, and meropenem was not observed; however, varying percentages of isolates are resistant to tetracycline, erythromycin, clindamycin, enrofloxacin, ciprofloxacin, amikacin, tiamulin, and lincomycin. Aminoglycosides are not recommended for intrinsic resistance [36], consistent with laboratory tests [5, 21, 51, 60]. MIC of tetracycline against the ZJ strain was 32 *μ*g/mL, in contrast to 4 *μ*g/mL against the ML101, as TetM is premature in the ML101. In line with other studies, threonine at 86 and aspartic acid at 90 in *gyrA* and serine at 81 in *parC* in the ML101 strain are sensitive to enrofloxacin with a cutoff value of 2 *μ*g/mL [5, 8].

Notably, (*aadE*-*apt*-*spw*-*lsa* (*E*)-*lnu* (*B*)-*aadE*-*sat4*–*aphA3*) in the chromosome of *E. rhusiopathiae* Ery-11 strain isolated in Sichuang China (2011–2014) was firstly reported by Zhang et al. [60] and subsequently identified in the ZJ strain isolated in Sichuang province in China in 2016 within the 77-kb insertion. More recently, four of 120 strains isolated in China from 2012 to 2018 were confirmed to contain the 77-kb insertion. Additionally, 37 of 40 strains in Hunan province in China were positive for this 77-kb insertion. This evidence demonstrated that the GI has been widely distributed in *E. rhusiopathiae* isolates since 2010. To date, whole genome with annotation of seven *E. rhusiopathiae* strains, B18, ZJ, GXBY-1, SE38, WH13013, SY1027, and ML101, isolated in diseased pigs in China from 2012 to 2018 have been determined. Genome-wide SNPs analysis and SpaA genotyping of these strains revealed that multiple strains were circulating in pig farms, and the Met203/Ile SpaA variant is predominant, similar to those found in Japan [6, 53].

The 77 kb GI was found in Chinese strains with an increasing tendency of distribution. Additionally, a 130-kb insertion containing a 77 kb sequence highly homologous to the GI found in ML101 was identified in a serotype 2 strain isolated from a domestic goose in Poland [61]. More importantly, the frequency of transfer of Tn*916* between *E. rhusiopathiae* strains was about tenfold higher than that between *E. faecalis* and *E. rhusiopathiae* [17], and subinhibitory concentrations of some ribosome-targeting antibiotics promote transfer of Tn*916* [62], which might be responsible for rapid spread of the GI among *E. rhusiopathiae* and raise the safety concerns of live attenuated vaccine. Additionally, residual virulence of live vaccines can cause chronic diseases in pigs, as demonstrated by bacterial isolation and molecular analysis [14].

Currently, live attenuated (G_4_T_10_ and GC42 strains), inactivated (CVCC43005 strain), and SpaA-based subunit vaccines (HIPRA) have been licensed in China. During 2019 in China, 79.7% of lot release of erysipelas vaccines were live attenuated, 11.4% were killed, and 8.9% were recombinant subunit according to China National Veterinary Drug Basic Information Database. In contrast, live attenuated (62.2%), inactivated (28.6%), and autogenous (9.2%) erysipelas vaccines were used in the USA [4]. Autogenous vaccines are also used to control swine infectious diseases in China, but no data is available as it is not officially encouraged.

Although the high virulence of ML101 with the GI was found to lead to outbreaks, the pathogenic mechanisms, especially their relationships with the GI, were not determined.

In summary, we identified a highly virulent *E. rhusiopathiae* strain, ML101, with a 77 kb GI. The MLD of the strain in mice and pigs is reportedly low; however, the mechanism underlying its high virulence requires further investigation. This work suggests that the re-emergence of erysipelas may be caused by new virulent strains, which contain a transmissible transposon carrying a multidrug resistance gene cluster. Therefore, subunit rather than live vaccines is recommended to prevent and control *E. rhusiopathiae* infection in pigs. Whether the GI contributes to the high virulence or horizontal transfer of virulence-associated genes remains to be investigated.

## Figures and Tables

**Table 1:** Summary of the macroscopic examination of piglets post-ML101 infection.

Challenge dose (CFU)	Pig no.	Clinical manifestations				Macroscopic changes						Mortality
		Depression	Partial or complete inappetence	Fever	Red or purple skin in hip	Auricle, epicardium, or myocardium blooding	Submandibular lymph node swelling or blooding	Spleen enlargement	Bubbles in the trachea and/or bronchus	Bladder hemorrhage	Mesenteric lymph node swelling or blooding	
1.20 × 10^9^	01	+	+	+	+	+	+	+	+	+	+	5/5
	02	+	+	+	+	+	+	+	−	−	+	
	03	+	+	+	−	+	−	+	+	+	+	
	04	+	+	+	+	+	+	+	−	−	+	
	05	+	+	+	+	+	+	+	+	+	+	

1.09 × 10^7^	06	+	+	+	+	+	+	+	+	−	+	5/5
	07	+	+	+	+	+	+	+	+	−	−	
	08	+	+	+	+	+	+	+	+	+	+	
	09	+	+	+	−	+	+	+	−	−	+	
	10	+	+	+	+	+	+	+	+	+	+	

1.05 × 10^5^	11	+	+	+	+	+	+	+	+	+	+	5/5
	12	+	+	+	+	+	+	+	−	−	+	
	13	+	+	+	−	+	+	+	+	+	−	
	14	+	+	+	+	+	+	+	−	−	+	
	15	+	+	+	−	+	+	+	+	+	+	

1.08 × 10^3^	16	+	+	+	+	+	+	+	−	+	+	5/5
	17	+	+	+	+	+	+	+	−	+	+	
	18	+	+	+	−	+	+	+	+	−	+	
	19	+	+	+	+	+	+	−	−	−	+	
	20	+	+	+	+	+	+	+	+	+	+	

1.0 × 10^1^	21	+	+	+	+	+	+	+	+	+	+	5/5
	22	+	+	+	+	+	+	+	−	−	+	
	23	+	+	+	+	+	+	−	+	−	+	
	24	+	+	+	−	+	+	+	−	−	+	
	25	+	+	+	+	+	+	+	−	+	+	

Blank control	26	−	−	−	−	−	−	−	−	−	−	0/5
	27	−	−	−	−	−	−	−	−	−	−	
	28	−	−	−	−	−	−	−	−	−	−	
	29	−	−	−	−	−	−	−	−	−	−	
	30	−	−	−	−	−	−	−	−	−	−	

**Table 2:** SpaA genotyping of *E. rhusiopathiae* strains.

Strain	Codon (aa 55)	Codon (aa 70)	Codon (aa 101)	Codon (aa 178)	Codon (aa 195)	Codon (aa 203)	Codon (aa 257)	Codon (aa 303)	
ML101	GTA (Val)	AAA (Lys)	AAC (Asn)	GGT (Gly)	GAT (Asp)	ATG (Met)	ATT (Ile)	GGG (Gly)	Met203/Ile257, spaA gene is identical
SE38	GTA (Val)	AAA (Lys)	AAC (Asn)	GGT (Gly)	GAT (Asp)	ATG (Met)	ATT (Ile)	GGG (Gly)	
ZJ	GTA (Val)	AAA (Lys)	AAC (Asn)	GGT (Gly)	GAT (Asp)	ATG (Met)	ATT (Ile)	GGG (Gly)	
B18	GTA (Val)	AAA (Lys)	AAC (Asn)	GGT (Gly)	GAT (Asp)	ATG (Met)	ATT (Ile)	GGG (Gly)	
GXBY-1	GTA (Val)	AAA (Lys)	AAC (Asn)	GGT (Gly)	GAT (Asp)	ATG (Met)	ATT (Ile)	GGG (Gly)	
WH13013	GTA (Val)	AAA (Lys)	AAC (Asn)	GGT (Gly)	GAT (Asp)	ATG (Met)	ATT (Ile)	GGG (Gly)	
SY1027	GTA (Val)	AAA (Lys)	AAC (Asn)	GGT (Gly)	GCT (Ala)	ATT (Ile)	ATT (Ile)	GGG (Gly)	Ile257
G4T10	GTA (Val)	AAA (Lys)	AAC (Asn)	GGT (Gly)	GAT (Asp)	ATT (Ile)	ATT (Ile)	GGG (Gly)	Ile257
C43065	GTA (Val)	AAA (Lys)	AAC (Asn)	GGT (Gly)	GAT (Asp)	ATT (Ile)	CTT (Leu)	GGG (Gly)	—
Fujisawa	GTA (Val)	AAA (Lys)	AAC (Asn)	GGT (Gly)	GAT (Asp)	ATT (Ile)	CTT (Leu)	GGG (Gly)	—
NCTC8136	ATA (Ile)	AAT (Asn)	AAC (Asn)	GAT (Asp)	AAT (Asn)	ATT (Ile)	ATT (Ile)	GAG (Gln)	—

**Table 3:** Insertions and deletions in the ML101 compared with the G_4_T_10_ and SE38.

Position	Strain	Length (bp)	Locus tag in ML101	Annotation	G4T10	SE38	ML101
470,479	G4T10	1,060	—	IS30-like element ISErh2 family transposase	+	−	−
1,492,198	G4T10	1,066	—	IS30-like element ISErh2 family transposase	+	−	−
1,485,081	G4T10	247	—	IS30-like element ISErh2 family transposase	+	−	−
1,254,199	SE38	1,364	—	IS3-like element ISErh1 family transposase	−	+	−
84,049	ML101	5,436	DM789_RS00395, DM789_RS00400, and DM789_RS00405	16 S, 23 S, and 5S rRNA	−	+	+
109,246	ML101	123	DM789_RS00505	SpaA	+, 2 20-aa repeat domain deletion	+	+
221,035	ML101	257	DM789_RS01030	tRNA-Gly	−	+	+
252,377	ML101	1,073	DM789_RS01195	IS30-like element ISErh4 family transposase	−	−	+
602,228	ML101	51	DM789_RS02905	Discoidin domain-containing protein	+	+	+, C-terminal PEVKPEEKPEVKPEEK insertion
875,062	ML101	194	DM789_RS04185	Flp family type IVb pillin	−	+	+
933,355	ML101	291	DM789_RS04480	DNA polymerase IV	+, premature at aa 117	+	+
1,014,404	ML101	1,062	DM789_RS04875	IS30-like element ISErh4 family transposase	−	−	+
1,337,145	ML101	77,233	—	GI	−	−	+

## Data Availability

The datasets presented in this study can be found in online repositories. The name of the repository and accession number can be found below: https://www.ncbi.nlm.nih.gov/nuccore/, CP029804.

## Supplementary Materials

Supplementary 1 Supplementary material contains Table S1–S7, and the table captions are listed as follows: Supplementary 1. Table 1: detection of GI in clinical *E. rhusiopathiae* and reference strains. Table 2: primer sets used in this study. Table 3: *E. rhusiopathiae* isolates used for whole genome-wide single-nucleotide polymorphisms analysis. Table 4: identification of the ML101 strain by biochemical tests. Table 5: MICs of antibiotics against the ML101. Table 6: determination of minimal lethal dose in mice. Table 7: virulence genes found in the ML101 genome. Figure 1: bacterial isolation of the clinical sample with sheep blood agar. The tissue sample was streaked on sheep blood agar followed by culture at 37°C for 48 hr. Figure 2: growth curve of the ML101 strain. The growth curve of the ML101 was determined by inoculation of 1% of overnight culture into fresh TSB medium supplemented with bovine serum followed by bacterial counting on TSA agar after tenfold serial dilutions at a 2-hr interval. Figure 3: gram stain of the ML101 strain. Fresh culture was stained with Gram kit (Hangzhou Microbial Reagent, Hangzhou, China) according to the manufacturer's instructions followed by examination under a microscopy; the magnification is 1,000-fold. Figure 4: PCR amplication to identify the ML101. A, 16S rDNA was ampified with primers 104-102, and 1177–1197 followed by Sanger sequencing (left panel); B, serotype 1a-specific amplification with primers 1a-F and 1a-R (right panel). Figure 5: serotyping assay by agar diffusion. Well #1, serotype 1a-specific serum; well #2, serotype 1a antigen control (G4T10 strain); well #3, ML101 strain; well #4, serotype 2 antigen control (CVCC43005 strain); well #5, PBS; and well #6, blank control. Precipitation line can be observed between well #1 and well #2 and between well #1 and well #3.

Supplementary 2 Sheet 1: nucleotide BLSAT analysis of the 16S rRNA amplified from the ML101 genome.

Supplementary 3 Sheet 2: genome-wide SNPs analysis among the ML101, G_4_T_10_, and SE38 strains.

Supplementary 4 Sheet 3: nucleotide BLAST analysis of the GI excluding Tn*916* and plasmid pEf37BA sequences in the ML101.

Supplementary 5 Sheet 4: genome-wide SNPs among the ML101, Fujisawa, SY1027, B18, G4T10, ZJ, WH13013, SE38, and GXBY-1.
