# Supplementary material for: Phenotypic and Genotypic Characterization of a Highly Virulent Erysipelothrix rhusiopathiae Strain
Source: Transbound Emerg Dis. 2024 Jul 26;2024:5401707. doi: 10.1155/2024/5401707 (PMC12017102; doi:10.1155/2024/5401707)
Supplement: Supplementary 1 — Supplementary material contains Table S1–S7, and the table captions are listed as follows: Supplementary 1. Table 1: detection of GI in clinical E. rhusiopathiae and reference strains. Table 2: primer sets used in this study. Table 3: E. rhusiopathiae isolates used for whole genome-wide single-nucleotide polymorphisms analysis. Table 4: identification of the ML101 strain by biochemical tests. Table 5: MICs of antibiotics against the ML101. Table 6: determination of minimal lethal dose in mice. Table 7: virulence genes found in the ML101 genome. Figure 1: bacterial isolation of the clinical sample with sheep blood agar. The tissue sample was streaked on sheep blood agar followed by culture at 37°C for 48 hr. Figure 2: growth curve of the ML101 strain. The growth curve of the ML101 was determined by inoculation of 1% of overnight culture into fresh TSB medium supplemented with bovine serum followed by bacterial counting on TSA agar after tenfold serial dilutions at a 2-hr interval. Figure 3: gram stain of the ML101 strain. Fresh culture was stained with Gram kit (Hangzhou Microbial Reagent, Hangzhou, China) according to the manufacturer's instructions followed by examination under a microscopy; the magnification is 1,000-fold. Figure 4: PCR amplication to identify the ML101. A, 16S rDNA was ampified with primers 104-102, and 1177–1197 followed by Sanger sequencing (left panel); B, serotype 1a-specific amplification with primers 1a-F and 1a-R (right panel). Figure 5: serotyping assay by agar diffusion. Well #1, serotype 1a-specific serum; well #2, serotype 1a antigen control (G4T10 strain); well #3, ML101 strain; well #4, serotype 2 antigen control (CVCC43005 strain); well #5, PBS; and well #6, blank control. Precipitation line can be observed between well #1 and well #2 and between well #1 and well #3. [file 5401707.f1.pdf]

Table S1. Detection of GI in clinical *E. rhusiopathiae* and reference strains

No.	Strain	Year of isolation	Location of isolation	Serotype	GI	Lesion	Morbidity	Mortality
1	ML101	2010	Miluo city	1a	+	Acute erysipelas	Unknown	17.1% (454/2650)
2	20100805	2010	Chenzhou city	1a	+	Acute erysipelas	Unknown	3.8% (29/748)
3	20100918	2010	Yueyang city	1a	+	Acute erysipelas	Unknown	5.1% (25/590)
4	20120612	2012	Huaihua city	1a	+	Acute erysipelas	Unknown	Unknown
5	20120615	2012	Changde city	1a	+	Subacute erysipelas	Unknown	1% (3/287)
6	20120726	2012	Yongzhou city	1a	+	Acute erysipelas	Unknown	Unknown
7	20120823	2012	Hengyang city	1a	+	Acute erysipelas	Unknown	Unknown
8	20130419	2013	Shaoyang city	1a	+	Acute erysipelas	Unknown	1% (2/177)
9	20130910	2013	Chenzhou city	1a	+	Acute erysipelas	Unknown	3% (27/892)
10	20130919	2013	Chenzhou city	1a	+	Acute erysipelas	Unknown	Unknown
11	20131220	2013	Yueyang city	1a	+	Acute erysipelas	Unknown	Unknown
12	20140128	2014	Xiangtan city	1a	+	Acute erysipelas	Unknown	Unknown
13	20140318	2014	Hengyang city	1a	-	Acute erysipelas	Unknown	Unknown
14	20140416	2014	Yiyang city	1a	+	Acute erysipelas	Unknown	1.0% (3/280)
15	20140628	2014	Chenzhou city	1a	+	Acute erysipelas	Unknown	Unknown
16	20140114	2014	Chenzhou city	1a	+	Acute erysipelas	Unknown	1.3% (5/390)
17	WSC	2014	Guangdong province	2	-	Subacute erysipelas	Unknown	Unknown
18	2014WSGY	2014	Guangdong province	1a	+	Subacute erysipelas	Unknown	Unknown
19	2014WSSC	2014	Guangdong province	2	-	Subacute erysipelas	Unknown	Unknown
20	2014WSSM	2014	Yiyang city	1a	+	Acute erysipelas	Unknown	Unknown

21	20170809	2017	Changde city	1a	+	Acute erysipelas	Unknown	Unknown
22	20171012	2017	Chenzhou city	1a	+	Acute erysipelas	Unknown	2.1% (6/290)
23	20180508	2018	Chenzhou city	1a	+	Acute erysipelas	Unknown	Unknown
24	20180801	2018	Jiangxi province	1a	+	Acute erysipelas	Unknown	0.4% (4/930)
25	20180802	2018	Yiyang city	1a	+	Acute erysipelas	Unknown	3.2% (6/190)
26	20190624	2019	Yueyang city	1a	+	Acute erysipelas	Unknown	0.7% (13/1890)
27	20190726	2019	Ningxiang city	1a	+	Acute erysipelas	Unknown	0.2% (5/2130)
28	20200817	2020	Yueyang city	1a	+	Acute erysipelas	Unknown	0.4% (7/1620)
29	20201229	2020	Yueyang city	1a	+	Acute erysipelas	Unknown	Unknown
30	20210701	2021	Chenzhou city	1a	+	Acute erysipelas	Unknown	2.6% (7/270)
31	20210908	2021	Hengyang city	1a	+	Acute erysipelas	Unknown	0.4% (5/1300)
32	20210913	2021	Chenzhou city	1a	+	Acute erysipelas	Unknown	0.3% (3/1080)
33	20220521	2022	Jiangxi province	1a	+	Acute erysipelas	Unknown	1.8% (9/490)
34	20220814	2022	Yueyang city	1a	+	Acute erysipelas	Unknown	Unknown
35	20220817	2022	Loudi city	1a	+	Acute erysipelas	Unknown	1% (5/450)
36	20220828	2022	Loudi city	1a	+	Acute erysipelas	Unknown	Unknown
37	20220927	2022	Shaoyang city	1a	+	Acute erysipelas	Unknown	0.5% (7/1450)
38	20230516	2023	Chenzhou city	1a	+	Acute erysipelas	Unknown	0.5% (3/540)
39	20230613	2023	Yueyang city	1a	+	Acute erysipelas	Unknown	0.8% (16/1870)
40	20230712	2023	Jiangxi province	1a	+	Acute erysipelas	Unknown	0.6% (7/1200)
41	C43-6	Before 1985	Guangdong province	2	-	Acute erysipelas (potency evaluation for vaccine development in China)	≥80% with high dose challenge	Unknown
42	C43-8	Before 1985	Hunan province	1a	-	Acute erysipelas (potency evaluation for vaccine	≥80% with high dose challenge	Unknown

				development in China)		
43	G_4T_{10}	1a	-	Avirulent vaccine strain	Unknown	Unknown
44	GC42	1a	-	None (vaccine strain)	Unknown	Unknown

Table S2. Primer sets used in this study

Primer name	Sequence (5'-3')		Annealing temperature of primer sets	Size of PCR product	Location of amplicon in the ML101 genome
1a-F<br>1a-R	ctcctaacgctttagcacgc<br>tgatcctttgccactaatgc	*E. rhusiopathiae* serotype 1a identification	60°C	356 bp	
104-120<br>1177-1197	ggcgvacgggtgagtaa<br>ccwttgyagcscgcgtgt	16S rDNA amplification	55°C	1094 bp	
ER-1F<br>ER-1R	gttcatctctctaatgcactac<br>tgttggactactaatcgtttcg	*E. rhusiopathiae* detection	58°C	399 bp	
PCV2F1<br>PCV2R1	accagcgcacttcggcagcggcag<br>gcgggccaaaaaaggtacagttcc	PCV2 detection	65°C	797 bp	
Outer primer F<br>Outer primer R	agrccagactggtggccntayga<br>ttyaccacttctgttctca	Nested PCR for CSFV detection (outer primers)	55°C	671 bp	
Inner primer F<br>Inner primer R	tcrwcaaccaaygagataggg<br>cacagyaaraayccraagtcatc	Nested PCR for CSFV detection (inner primers)	55°C	272 bp	
PRV-F<br>PRV-R	atgcggccctttctg<br>cggttctcccggtatttaagc	PRV detection	65°C	455 bp	
Nsp2-F<br>Nsp2-R	ctccgtggtgcaacaa<br>ggcttgagctgagtat	PRRSV detection	57°C	1146 bp	

1F	GCCTGGTCTTTCCAGGTCT	FMDV detection	55°C	328 bp	
1R	CCAGTCCCCTTCTCAGATC				
F1	ctttagtcttcccagtcattgtgc	SAM-dependent DNA methyltransferase encoding gene detection	59°C	503 bp	1343892-1344394
R1	atctgcgaaggaaacgaagtgt				
F2	caacaataaaaacaaccacacttc	cytoplasmic protein and its downstream hypothetical protein encoding gene detection	59°C	428 bp	1421233-1421660
R2	agactatttagggcgcttttatg				
F3	acgaaattggaatggagttatg	Enonuclease encoding gene detection	51°C	432 bp	1354774-1355205
R3	tatggcgaaaagcaatgtgt				
F4	actctaaatgggacgcaaaaat	Recombinase encoding gene detection	55°C	412 bp	1348068-1348479
R4	gcaaaaagagtcatatccagtaga				
F5	tcaaaactcgaagaaatcataaag	ATP-binding protein encoding gene detection	57°C	441 bp	1353539-1353979
R5	tctcagcaataggttctccatca				

The PCR cycling conditions were 5 min at 94 °C, followed by 30 cycles of 30 s at 94 °C, 30 s at annealing temperature of each primer set and 90 s at 72 °C. Final extension was done at 72 °C for 7 min. R=A/G, Y=C/T, N=A/T/C/G, W=A/T.

Table S3. *E. rhusiopathiae* isolates used for whole genome-wide single nucleotide polymorphisms analysis

Strain	Genome size (bp)	Accession No.	Location	Year of isolation
B18	1,931,527	NZ_CP080398	Hubei, China	2018
ZJ	1,945,689	NZ_CP041995	Sichuan, China	2016
GXBY-1	1,876,490	NZ_CP014861	Guangxi, China	2012
SE38	1,778,134	NZ_CP011861	China	2013
G4T10	1,770,505	NZ_CP011860	China	2013
WH13013	1,778,058	NZ_CP017116	China	2013
SY1027	1,752,910	CP005079	Jiangsu, China	2014
Fujisawa	1,787,941	NC_015601	Japan	Before 1985

Table S4. Identification of the ML101 strain by biochemical tests

Sugar fermentation	Result in this study
glucose	+
lactose	+
galactose	+
maltose	+
mannitol	+
sucrose	-
arabinose	-
xylose	-
salicin	-
fructose	-
nitrate	+
H2S	+
Voges−Proskauer	-
urease	-
indole	-
citrate	-
oxidase	-
methyl red	-
indole	-

ND: not determined.

Table S5. MICs of antibiotics against the ML101

Antibiotics	Breakpoints for sensitive	MICs (µg/mL)	Determination	Reference
Ampicillin	≤0.25	0.06	S	CLSI VET06
Penicillin	≤0.12	<0.015	S	CLSI VET06

Enrofloxacin	≤0.25	1	I	CLSI VET06
Amoxicillin	N.D	<0.015	N.D	
Levofloxacin	N.D	0.5	N.D	
Florfenicol	N.D	1	N.D	
Streptomycin	N.D	>512	N.D	
Tetracycline	N.D	4	N.D	
Vancomycin	N.D	8	N.D	
Ceftriaxone	N.D	<0.015	N.D	

Abbreviations: R, resistance; S, sensitive; I, intermediate; N.D, not determined.

Table S6. Determination of minimal lethal dose in mice

Challenge dose/CFU	Mortality
1,000	10/10
100	10/10
10	10/10
5	8/10
0 (blank control)	0/10

Table S7. Virulence genes found in the ML101 genome

**Locus tag**	**Functional annotation**
**Surface proteins**	
ERH_0075	Collagen-binding protein
ERH_0094	Protective antigen (spaA.1)
ERH_0150	Hyaluronidase (hylA)
ERH_0161	Peptidase M14
ERH_0201	Pectin lyase fold-containing protein
ERH_0221	Glycoside hydrolase, family 16
ERH_0260	Proteinase
ERH_0278	Unknown
ERH_0299	Neuraminidase (nanH.1)
ERH_0407	Choline-binding protein (cbpA)
ERH_0561	Glycosyl hydrolase, family 85
ERH_0668	Biofilm formation, protective antigen (rspA)
ERH_0728	Internalin-like
ERH_0765	Hyaluronidase (hylB)
ERH_0768	Adhesin, Plasminogenbinding protein (cbpB)
ERH_0777	Dipeptidase
ERH_1139	5 –Nucleotidase (ushA)
ERH_1210	Hyaluronidase (hylC)
ERH_1258	Unknown
ERH_1436	Collagen-binding protein
ERH_1454	Unknown
ERH_1472	Internalin-like
ERH_1687	Biofilm formation (rspC)
GAPDH	Glyceraldehyde-3-phosphate dehydrogenase

HP0728	
HP1472	
**Antioxidant proteins**	
ERH_0162	Thiol peroxidase (tpx)
ERH_0175	Alkylhydroperoxide reductase (ahpC)
ERH_0356	Glutaredoxin (nrdH)
ERH_0375	Thioredoxin (trxA.1)
ERH_1065	Superoxide dismutase (sodA)
ERH_1311	Thioredoxindisulfide reductase (trxB.1)
ERH_1345	Alkylhydroperoxide reductase (ahpD)
ERH_1500	Thioredoxin (trxA.2)
ERH_1541	Thioredoxindisulfide reductase (trxB.2)
**Phospholipase**	
ERH_0072	Patatin-like phospholipase
ERH_0083	Phospholipase/ Carboxylesterase family
ERH_0148	Lysophospholipase (pldB)
ERH_0333	Cardiolipin synthetase (cls)
ERH_0334	Patatin-like phospholipase
ERH_0388	Phospholipase D
ERH_1214	Lysophospholipase
ERH_1433	Lysophospholipase
**Hemolysins**	
ERH_0467	Hemolysin-related protein
ERH_0649	Hemolysin III
ERH_0761	Neuraminidase (nanH.2)
ERH_1034	Fibronectinbinding protein
ERH_1356	Adhesin
ERH_1467	Biofilm formation
**Regulator**	
ERH_0661	XRE family transcriptional regulator

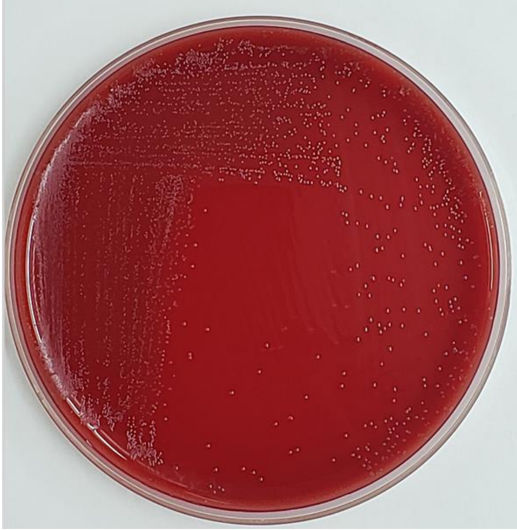

Figure S1. Bacterial isolation of the clinical sample with sheep blood agar. The tissue sample was streaked on sheep blood agar followed by culture at 37°C for 48 h.

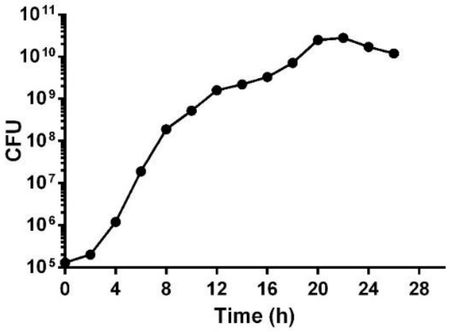

Figure S2. Growth curve of the ML101 strain. The growth curve of the ML101 was determined by inoculation of 1% of overnight culture into fresh TSB medium supplemented with bovine serum followed by bacterial counting on TSA agar after 10-fold serial dilutions at a 2 h-interval.

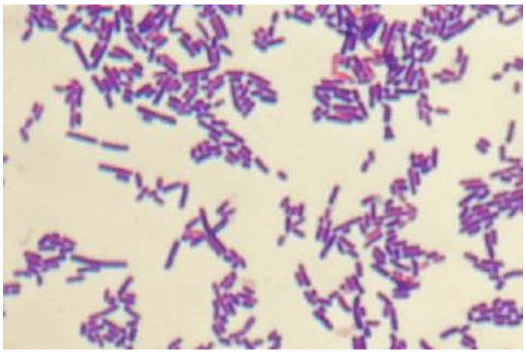

Figure S3. Gram-stain of the ML101 strain. Fresh culture was stained with Gram kit (Hangzhou microbial reagent, Hangzhou, China) according to the manufacturer's instructions followed by examination under a microscopy; the magnification is 1,000 fold.

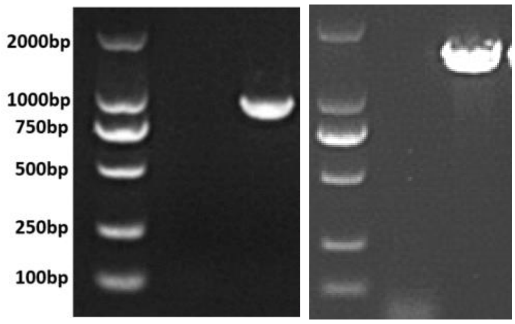

Figure S4. PCR amplication to identify the ML101. A, 16S rDNA was ampified with primers 104-102 and 1177-1197 followed by Sanger sequencing (left panel); B, serotype 1a-specific amplification with primers 1a-F and 1a-R (right panel).

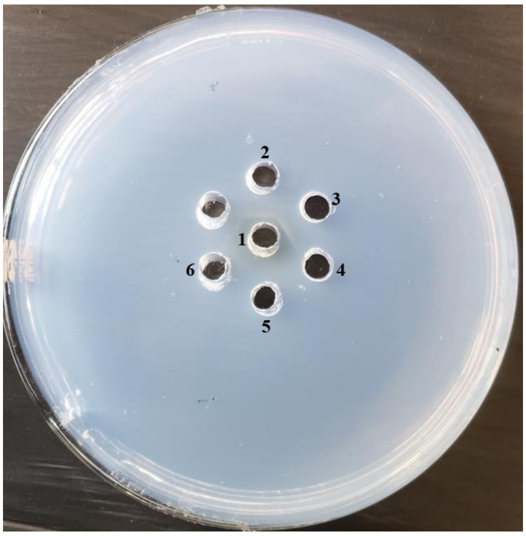

Figure S5. Serotyping assay by agar diffusion. Well #1, serotype 1a-specific serum; well #2, serotype 1a antigen control (G_4T_{10} strain) ; well #3, ML101 strain; well #4, serotype 2 antigen control (CVCC43005 strain); well #5, PBS; well #6, blank control. Precipitation line can be observed between well #1 and well #2, and between well #1 and well #3.
